# Similarities in Mechanisms of Ovarian Cancer Metastasis and Brain Glioblastoma Multiforme Invasion Suggest Common Therapeutic Targets

**DOI:** 10.3390/cells14030171

**Published:** 2025-01-23

**Authors:** Gia A. Jackson, David Cory Adamson

**Affiliations:** Neurosurgery Section, Atlanta VA Healthcare System, School of Medicine, Mercer University, Georgia Neurosurgical Institute, Macon, GA 31207, USA; gia.a.jackson@live.mercer.edu

**Keywords:** epithelial-to-mesenchymal transition (EMT), ovarian cancer, glioblastoma multiforme (GBM), tumor progression, metastasis, invasion, comparative genomic analysis, cell plasticity, therapy resistance, TWIST1, SNAIL, ZEB1, PI3K/AKT pathway, TGF-β signaling, mesenchymal phenotype, regulatory networks, gene expression profiles, therapeutic targets

## Abstract

Epithelial-to-mesenchymal transition (EMT) is a critical process in malignant ovarian cancer metastasis. EMT involves the conversion of epithelial cells to mesenchymal cells, conferring enhanced migratory and invasive capabilities. Glioblastoma multiforme (GBM) is the most common malignant primary brain tumor and exhibits an aggressive invasive phenotype that mimics some steps of EMT but does not undergo true metastasis, i.e., the invasion of other organ systems. This study conducts a comparative genomic analysis of EMT in ovarian cancer and invasion in GBM—two malignancies characterized by poor prognosis and limited therapies. Investigating the molecular biology in ovarian cancer and GBM demonstrates shared mechanisms of tumor progression, such as similar genetic and molecular pathways influencing cell plasticity, invasion, and resistance to therapy. The comparative analysis reveals commonalities and differences in the regulatory networks and gene expression profiles associated with EMT and invasion in these cancers. Key findings include the identification of core EMT regulators, such as TWIST1, SNAIL, and ZEB1, which are upregulated in both ovarian cancer and GBM, promoting mesenchymal phenotypes and metastasis. Additionally, the analysis uncovers EMT-related pathways, such as the PI3K/AKT and TGF-β signaling, which are critical in both cancers but exhibit distinct regulatory dynamics. Understanding the intricacies of EMT in ovarian cancer and invasion in GBM provides valuable insights into their aggressive behavior and identifies potential common therapeutic targets. The findings stress the importance of targeting EMT/invasion transitions to develop effective treatments to halt progression and improve patient outcomes in these malignancies.

## 1. Introduction to Ovarian Cancer and Glioblastoma Multiforme

Epithelial-to-mesenchymal transition (EMT) is a dynamic process that plays a crucial role in embryonic development, wound healing, and most cancer progression types. In most cancers, EMT refers to the transformation of epithelial cells, which are typically stationary and adherent, into mesenchymal cells that exhibit increased migratory and invasive capabilities that allow tumor cells to metastasize, i.e., break away from a primary tumor, migrate through the extracellular matrix, invade blood vessels and lymphatic systems, travel to distant sites and make their way to distant organs where they proliferate into tumors. Malignant gliomas like glioblastoma multiforme (GBM) in the brain do not metastasize but stay within the brain organ. However, their cells demonstrate different phenotypes, including highly invasive and migratory capacities that allow them to travel to distant sites within the brain.

Ovarian cancer and GBM are two malignancies with poor prognoses and high mortality rates. The prevalence and impact of these cancers are significant. Ovarian cancer is the leading cause of death from gynecologic malignancies, characterized by a high degree of heterogeneity with several subtypes. The most common subtype of ovarian cancer is epithelial ovarian cancer (EOC), which accounts for about 90% of all ovarian cancers. EOC originates from the cells that cover the outer surface of the ovary, and there are several subtypes, including serous, mucinous, endometrioid, and clear cell carcinomas, with high-grade serous carcinoma (HGSC) being the most prevalent and the focus of our analysis. Ovarian cancer has a lifetime risk of approximately 1 in 78 women, with an estimated 22,000 new cases and 14,000 deaths annually in the United States alone [1]. The standard treatment for HGSC depends on factors such as the cancer’s stage and grade, the patient’s overall health, and specific tumor characteristics. Treatment typically involves a combination of surgery, to remove as much of the tumor as possible (debulking), and chemotherapy, often with platinum- and/or taxane-based drugs [2]. The median survival varies based on the stage at diagnosis, but for patients with advanced-stage HGSC, it is generally around two years, with many factors influencing individual outcomes [2,3]. GBM, the most common malignant primary brain tumor, is marked by rapid growth and a propensity for invasive behavior within the brain. It accounts for about 15% of all primary brain tumors. It has a median survival of only 12–15 months despite aggressive treatment modalities, including a maximally safe surgical resection, radiation, and nonspecific temozolomide chemotherapy [4,5]. Despite these interventions, both cancers exhibit resistance to conventional therapies. Thus, their prognoses remain poor, underscoring the urgent need for novel therapeutic strategies.

**Figure 1:**
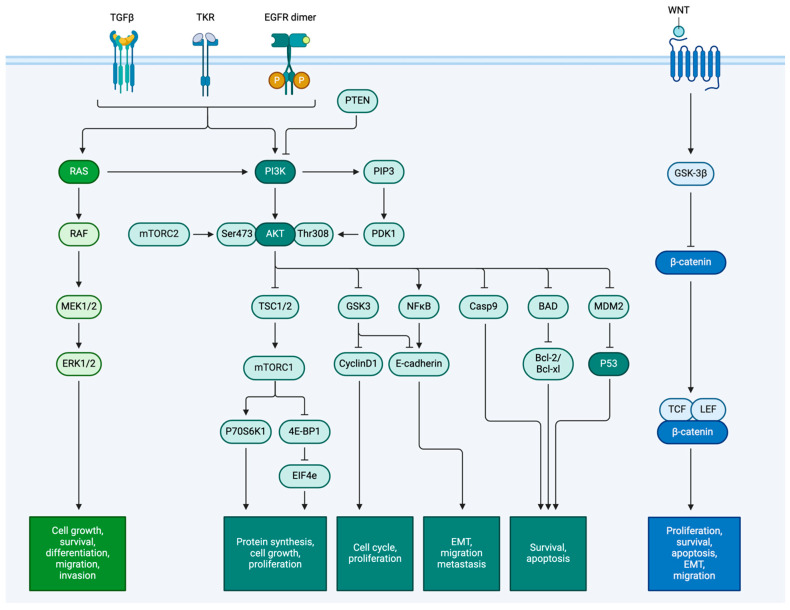
Commonly mutated signaling pathways driving both EMT in HGSC and invasion in GBM. The figure highlights key signaling pathways in cancer, particularly during EMT and invasion. Key players include TGFβ, EGFR, and Wnt. These pathways are activated when ligands bind to receptors, triggering a series of phosphorylation events. For example, the RAS/RAF/MEK/ERK pathway drives cell growth and movement, but mutations can cause uncontrolled proliferation and EMT. The PI3K/AKT/mTOR pathway promotes survival and growth; mutations keep AKT signaling active, aiding EMT and invasion. The Wnt pathway stabilizes β-catenin and affects cell proliferation and migration, with mutations increasing EMT and metastasis. TGFβ is crucial for EMT, enhancing cell invasion. These pathways work together to regulate cancer progression, with mutations making EMT and metastasis more likely [6].

Biologically, both ovarian cancer and GBM are characterized by complex genetic and molecular landscapes that promote tumor growth and progression. Key features include numerous genetic mutations, aberrant signaling pathways, and the capacity to undergo EMT and invasion, facilitating their proliferative capabilities. We describe the comparative genomic analysis of EMT in ovarian cancer and invasion in GBM and identify common and distinct regulatory networks and pathways that drive these transitions (Figure 1). By understanding the molecular framework of these processes in these cancers, we uncover potential therapeutic targets that could improve treatment outcomes and patient survival. This analysis provides insights into the invasive behavior of these malignancies and highlights avenues for developing more effective interventions.

## 2. Background for Ovarian Cancer EMT and GBM Invasion Mechanisms

EMT is a pivotal cellular plasticity process essential for various physiological and pathological conditions. EMT is characterized by the conversion of polarized, immotile epithelial cells into motile mesenchymal cells, which involves a loss of cell–cell adhesion and a gain of migratory and invasive properties [7]. This transition is also crucial during embryonic development, where it facilitates tissue and organ formation and plays a significant role in wound healing by enabling the movement of cells to sites of injury for tissue repair.

**Figure 2:**
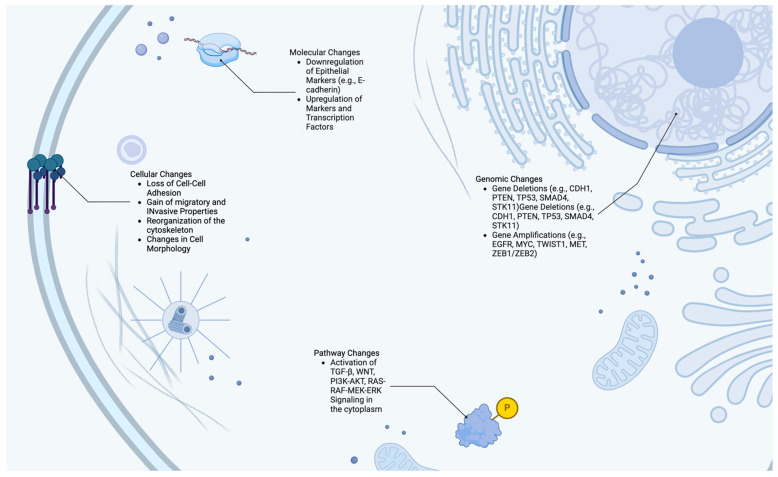
Key genomic, molecular, pathway, and cellular changes involved in EMT. This figure illustrates the key genomic, molecular, pathway, and cellular changes involved in EMT. It highlights genomic alterations, such as amplifications and deletions that drive EMT. The molecular section details the role of transcription factors like SNAIL, TWIST1, and ZEB1. As well as mesenchymal markers like N-cadherin and vimentin. The pathway segment depicts key signaling pathways, including TGF-β, Wnt, RAS-RAF-MEK-ERK, and PI3K-AKT, which regulate EMT. The cellular changes panel shows the morphological transformation from epithelial to mesenchymal phenotype, characterized by loss of cell–cell adhesion and increased migratory capacity [11].

At the molecular level, EMT is regulated by complex networks involving transcription factors, signaling pathways, and epigenetic modifications (Figure 2). Various growth factors, cytokines, and extracellular matrix (ECM) components influence the activation of EMT-related transcription factors, which orchestrate the phenotypic changes necessary for invasive behavior [8]. This transition not only facilitates local invasion by augmenting the penetration of the ECM but also enhances the cancer cell’s ability to survive in circulation, colonize new sites, and establish metastatic lesions by intravasating and then extravasating blood or lymphatic vessels. After reaching distant sites, a reverse process allows these cells to re-establish epithelial characteristics and form new colonies, contributing to metastatic outgrowth [9]. Genomic alterations in cancer disrupt standard regulatory mechanisms and promote EMT through mutations, the loss of epithelial genes, and amplifications of mesenchymal traits. Epigenetic modifications, such as DNA methylation and histone changes, further facilitate EMT by suppressing epithelial genes, activating mesenchymal programs, and promoting crucial transcription factors that drive the transition from epithelial to mesenchymal states, supporting various biological processes, including development, tissue repair, and disease progression [10]. Collectively, these mechanisms emphasize the interplay between genetic, epigenetic, and extracellular factors in driving EMT and metastasis in cancer.

Invasion is one of the hallmarks of cancer progression, crucial for the transition from localized tumors to more aggressive forms. Through the loss of cell–cell adhesion and secretion of proteolytic enzymes that degrade the ECM, cancer cells acquire the ability to invade surrounding tissues and gain access to blood or lymphatic vessels, possibly facilitating metastasis [7,12]. However, not all invasive tumors lead to metastasis. For instance, GBM invariably invades neighboring brain tissue but does not spread outside the central nervous system. The invasion process, like EMT, is regulated by complex signaling pathways and microenvironmental factors that promote the migratory and invasive behaviors of cancer cells. However, the two processes are not mutually exclusive; they are interconnected and often coexist during cancer progression [12].

The intricate regulation of EMT and invasion by multiple signaling pathways and genomic alterations indicates these processes’ complexity and significance in cancer progression. Evidence suggests that EMT is the critical event that makes cancer fatal, as studies have shown that while surgeons can often remove a single tumor, the process of EMT facilitates metastasis, significantly reducing the effectiveness of subsequent treatments. This transition allows cancer cells to acquire invasive and migratory properties, enabling them to spread to distant organs and establish secondary tumors that are more resistant to conventional therapies [8]. Variables such as hypoxia, cytokines, immune cells, and more within the tumor microenvironment (TME) can exacerbate EMT and invasion, promoting conditions that support cancer progression [13]. Understanding these interactions is crucial for developing interventions that effectively target these processes and improve patient outcomes.

## 3. Ovarian Cancer

### 3.1. Overview of Ovarian Cancer

Ovarian cancer is a heterogeneous disease with several subtypes, each characterized by distinct histopathological and molecular features. The diagnostic criterion for ovarian cancer involves a combination of clinical evaluation, imaging studies, laboratory tests, and histopathological examination [14]. Persistent symptoms like bloating, pelvic pain, and urinary urgency warrant further investigation through pelvic exams and imaging such as transvaginal ultrasound, CT, MRI, and PET scans. Elevated CA-125 levels may also suggest ovarian cancer, but ultimately, a biopsy is required for diagnosis [14].

The most common and aggressive form is HGSC, which accounts for approximately 70% of ovarian cancer cases [15]. Other malignant types include endometrioid, clear cell, and mucinous carcinomas, each with unique genomic landscapes and clinical behaviors (see Table 1 for a summary of genomic alterations in ovarian cancer). HGSC represents a challenge due to its poor prognosis. This subtype of ovarian cancer often presents at an advanced stage, contributing to its high mortality rate. The current standard treatment involves surgical debulking, followed by platinum-based chemotherapy [2,16]. Despite initial response rates, recurrence is common, highlighting the need for more effective therapeutic targets and strategies.

### 3.2. Genomic Alterations in Ovarian Cancer

The genomic landscape of ovarian cancer is marked by various alterations, including dysregulated pathways, mutations, copy number variations (CNVs), and chromosomal rearrangements (see Table 1). These alterations contribute to tumor initiation, progression, and metastasis. The primary pathways dysregulated in ovarian cancer include the DNA repair pathway, cell cycle regulation, and signaling pathways such as PI3K/AKT and RAS/MAPK. Critical components in these pathways are often mutated, contributing to cancer progression and poor prognosis. Mutations in TP53 are found in over 96% of HGSC cases, making it the most commonly altered gene in ovarian cancer. TP53 mutations lead to the loss of its tumor suppressor function, contributing to genomic instability and cancer progression [17,18]. Approximately 5–15% of ovarian cancers are associated with inherited BRCA1 or BRCA2 mutations, which not only increase cancer risk but also impact treatment responses, making tumors more susceptible to platinum-based chemotherapy and PARP inhibitors [16,19]. These mutations impair DNA repair via homologous recombination, increasing susceptibility to further genetic alterations, which lead to tumor progression. Another frequently seen mutation is in PIK3CA, which encodes a subunit of the PI3K enzyme and is frequent in endometrioid and clear cell carcinomas [17]. This pathway’s alteration includes PIK3CA mutations and a loss of PTEN function, occurring in approximately 12–20% of ovarian cancers [20]. These changes lead to uncontrolled cell proliferation and survival, contributing to tumor growth and resistance to apoptosis. The PI3K/AKT pathway alterations are also linked to resistance to conventional therapies, highlighting the need for targeted treatments in affected patients. The RAS/MAPK pathway is another signaling pathway often dysregulated in ovarian cancer, with mutations in KRAS observed in about 10–15% of cases [20]. These mutations lead to continuous pathway activation, promoting cell growth, division, and survival. Understanding these molecular alterations is crucial for developing targeted therapies and improving patient outcomes. Efforts to personalize treatment based on each tumor’s specific genetic and molecular profile are ongoing, aiming to enhance the effectiveness of therapeutic interventions and minimize side effects.

CNVs are prevalent in ovarian cancer, particularly in HGSC, where widespread copy number gains and losses affect numerous genes involved in cell cycle regulation, apoptosis, and DNA repair. For example, amplifications of CCNE1 (cyclin E1) via increased CNVs are associated with poor prognosis because they can cause replication stress and genomic instability. Deletions associated with CDKN2A/B cause loss of cell cycle regulation, leading to uncontrollable cell growth and tumor progression [15]. Chromosomal rearrangements, such as translocations and inversions, can lead to the formation of oncogenic fusion genes or the disruption of tumor suppressor genes, further driving tumorigenesis. For example, chromosome 7 gains often involve EGFR amplification, while chromosome 10 losses involve PTEN loss, contributing to tumorigenesis. EGFR amplification and PTEN loss play critical roles in ovarian cancer progression by regulating key signaling pathways involved in cell proliferation, survival, and tumor growth by enhancing downstream signaling pathways such as PI3K-AKT. Understanding the complex interactions and impacts of these genetic alterations is crucial for developing targeted therapeutic strategies to improve patient outcomes in ovarian cancer.

### 3.3. EMT in Ovarian Cancer

EMT plays a crucial role in the progression and metastasis of ovarian cancer. Genetic alterations such as mutations and CNVs in genes like TP53, BRCA1 and 2, and those in the PI3K/AKT pathway can induce EMT (Figure 1). For example, mutations in TP53 are associated with an increased expression of EMT markers [17]. P53 is the tumor suppressor protein that TP53 encodes. When TP53 is mutated, the mutant protein product has the ability to modulate the TGF-β pathway, Snail (SNA1) and Slug (SNA2) expression, and microRNA responsible for EMT induction [18]. Under normal conditions, TGF-β signaling inhibits cellular proliferation and induces programmed cell death. However, this pathway can shift in cancer to support EMT and metastasis. The mutant P53 protein produced can enhance this shift, promoting cancer progression.

Typically, BRCA1 and 2 are critical in repairing damaged DNA through homologous recombination to provide genomic stability. However, when these genes are mutated, the repair process is compromised, leading to the buildup of damage and mutations in DNA. This accumulation of damage leads to genomic instability that can initiate pathways that induce EMT of the affected cells and facilitate cancer progression. As seen with TP53, the genomic instability caused by BRCA1 and 2 mutations can similarly increase the expression of the TGF-β signaling pathway, Snail, Slug, and microRNAs that enhance tumor invasiveness and metastatic potential.

Changes in the PI3K/AKT pathway also trigger EMT in ovarian cancer. Under normal conditions, this pathway’s function is the regulation of cell growth, survival, and metabolism. However, when PI3K is mutated, or PTEN is lost, the pathway becomes constantly activated, driving uncontrolled cell growth by ignoring typical regulation (Figure 1). Like the other genes mentioned above, this constitutive activation increases transcription factors such as Snail, Slug, and Twist1. Snail and Slug are transcription factors that repress E-cadherin expression, promoting the loss of cell–cell adhesion and facilitating the transition to a mesenchymal phenotype [21]. Twist1 is another transcription factor known to induce EMT by promoting the expression of mesenchymal markers and enhancing cell motility, thereby facilitating the invasive behavior of cancer cells [21]. Additionally, epigenetic modifications such as DNA methylation and histone modifications can silence epithelial markers (e.g., E-cadherin) and activate mesenchymal markers (e.g., N-cadherin, vimentin), further promoting the transition to a mesenchymal phenotype. These transcription factors play crucial roles in driving the molecular changes that underlie the metastatic potential of ovarian cancer cells, making them important targets for therapeutic intervention strategies aimed at inhibiting metastasis.

### 3.4. Role of Tumor Microenvironment in Ovarian Cancer

**Figure 3:**
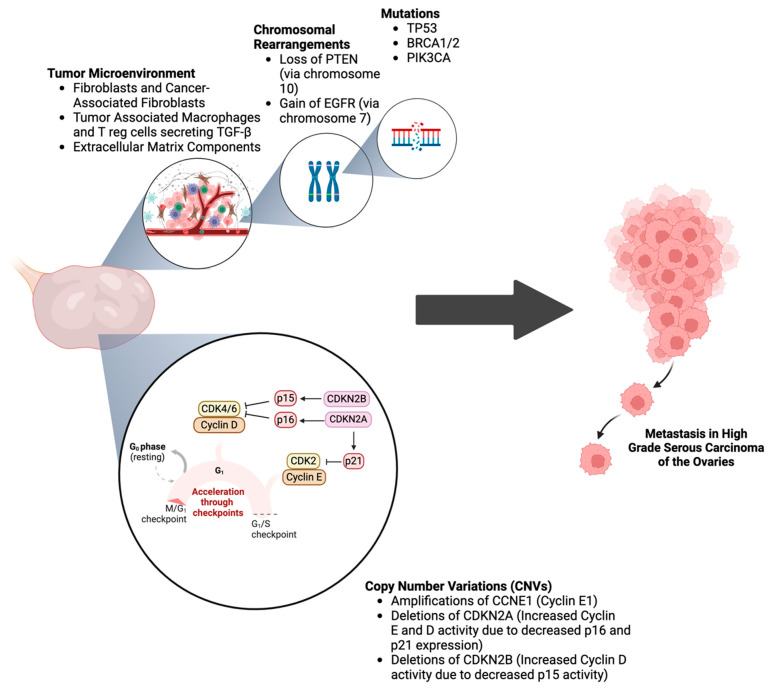
**The genomic landscape of ovarian cancer.** This figure presents the genomic landscape of ovarian cancer, highlighting key features of the tumor microenvironment, chromosomal rearrangements, mutations, and CNVs. The tumor microenvironment section shows interactions between cancer cells, stromal cells, immune cells, and the extracellular matrix. The chromosomal rearrangements panel displays common structural alterations like translocations and inversions. The mutations section details frequently mutated genes such as TP53, BRCA1, and BRCA2. Lastly, the CNVs panel highlights genomic amplification and deletion regions, affecting critical oncogenes and tumor suppressor genes [24].

The TME in ovarian cancer significantly influences EMT (Figure 3). Components such as cancer-associated fibroblasts (CAFs), immune cells, and ECM components secrete cytokines and growth factors that can induce EMT in cancer cells. The drastic differences in the microenvironment between ovarian cancer and other malignancies, such as GBM, lead to tissue-specific outcomes. For example, TGF-β, a cytokine abundant in the TME, is a potent inducer of EMT due to its immunosuppressive function [22]. The interaction between ovarian cancer cells and the TME creates a supportive niche for cancer progression and metastasis. Moreover, the dynamic interplay of the cancer cells and stromal cells of the TME facilitates the production of a pro-inflammatory environment that is essential for cancer initiation, progression, metastasis, and therapeutic resistance. Specifically, CAFs play a critical role by producing collagen and fibronectin, remodeling the ECM to promote tumor cell migration and invasion. Simultaneously, CAF-derived cytokines such as IL-6 and IL-8 stimulate EMT pathways and contribute to tumor growth and chemoresistance [23].

The immune components of the ovarian cancer microenvironment further exacerbate disease progression. Tumor-associated macrophages (TAMs), myeloid-derived suppressor cells (MDSCs), and regulatory T cells (Tregs) secrete immunosuppressive cytokines, including IL-10 and TGF-β, that dampen anti-tumor immunity and enable immune evasion [23,25,26]. This immunosuppressive environment creates favorable conditions for tumor progression by facilitating cellular plasticity and altering the tumor microenvironment. TAMs polarize into an M2-like phenotype that enhances EMT, angiogenesis, and metastasis through the secretion of VEGF and matrix metalloproteinases (MMPs), which degrade the ECM and promote tissue invasion [25,26].

Moreover, the unique features of the ovarian cancer microenvironment, including peritoneal fluid dynamics, create a permissive environment for the widespread dissemination of tumor cells within the abdominal cavity. The accumulation of ascites, which contains tumor cells, CAFs, immune cells, and soluble factors, acts as a reservoir for pro-inflammatory cytokines, chemokines, and growth factors that further drive EMT, tumor progression, and resistance to therapy [26]. The dynamic crosstalk between cancer cells and the ascitic microenvironment accelerates metastasis and enhances tumor cell survival and immune evasion.

Understanding the complexity of the ovarian cancer TME and its role in driving EMT is essential for identifying novel therapeutic targets. While therapies targeting TGF-β and VEGF pathways have shown promise in preclinical studies, their clinical efficacy remains limited, emphasizing the need for alternative approaches, e.g., combinatorial approaches [26,27,28]. Disrupting the interaction between cancer cells and the stromal or immune components of the TME, such as blocking CAF-derived cytokines or reprogramming TAMs, may provide practical strategies to inhibit EMT, reduce metastatic burden, and overcome therapeutic resistance. Targeting the ECM components that support cancer cell migration and invasion, such as collagen and fibronectin, could be another avenue to enhance the efficacy of existing therapies by providing synergistic effects when combined with other treatments. Moreover, targeting the immune microenvironments to reverse immune suppression could improve the response to conventional therapies and restore the body’s ability to fight off cancer. As our understanding of the TME deepens, the identification of additional key regulators of EMT, such as specific cytokines or metabolic factors, will be crucial in developing more precise and effective therapeutic strategies for ovarian cancer.

## 4. Glioblastoma Multiforme (GBM)

### 4.1. Overview of GBM

GBM is the most common malignant primary brain tumor in adults. It is classified as grade IV astrocytoma by the World Health Organization (WHO) and is characterized by rapid growth, invasiveness, and a highly heterogeneous genetic composition [3,27]. GBM can be classified into primary (de novo) and secondary types based on clinical and molecular features. Primary GBM arises de novo without evidence of a precursor lower-grade glioma and is more common in older adults. Secondary GBM develops from lower-grade glioma and tends to occur in younger patients. Primary GBM accounts for approximately 90% of cases [28]. Epidemiologically, GBM has an incidence of 3.19 cases per 100,000 person-years in the United States. It affects men slightly more than women and is more prevalent in older adults, with the median age at diagnosis being 64 years [29].

**Figure 4:**
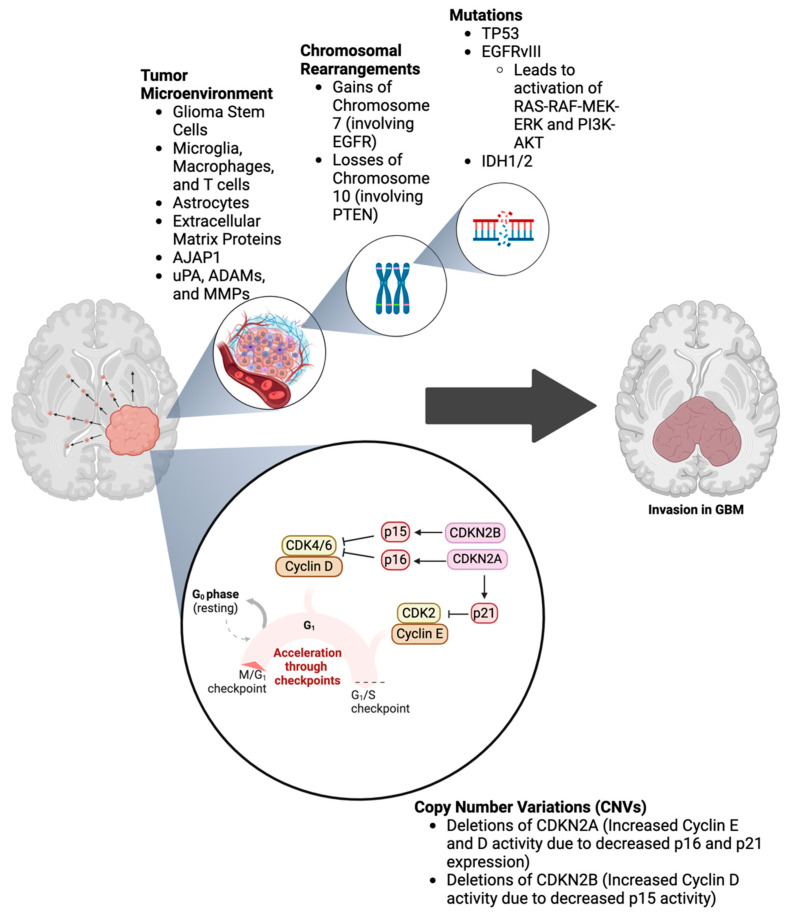
**Genomic landscape of GBM.** This figure illustrates the genomic landscape of GBM, highlighting the tumor microenvironment, chromosomal rearrangements, mutations, and CNVs. The tumor microenvironment section shows interactions among cancer cells, stromal cells, stem cells, immune cells, and the extracellular matrix with AJAP1, uPA, ADAMs, and various MMPs in facilitating tumor invasion and progression. The chromosomal rearrangements panel highlights gains and losses involving key genes such as EGFR and PTEN. The mutations section details altered genes, including TP53, EGFRvIII, and IDH1/2, in GBM pathogenesis. The CNVs panel shows genomic amplification and deletion regions, affecting crucial oncogenes and tumor suppressor genes [30].

The 2021 WHO diagnostic criteria for GBM incorporate histological and molecular features for precise classification. Histologically, the presence of necrosis and/or microvascular proliferation is necessary. Molecularly, most primary GBMs lack mutations in isocitrate dehydrogenase (IDH) genes that differentiate them from lower-grade gliomas and secondary GBMs with IDH mutations [3,5,27]. The genomic landscape of GBM, as illustrated in Figure 4 and summarized in Table 2, is highly heterogeneous and includes mutations in key genes such as TP53, EGFR, and IDH1/2, which drive tumorigenesis and progression. Other standard molecular features in GBM include TERT promoter mutations and EGFR gene amplification, without 1p/19q codeletion, and additional markers such as TP53 mutations, which are more common in secondary GBMs [3].

Despite advances in diagnostic criteria and treatment, the prognosis for GBM remains poor, with a median survival time of approximately 15 months following diagnosis. Current standard treatment involves maximal surgical resection followed by concurrent radiotherapy and temozolomide chemotherapy [5]. Even with these multimodal approaches, GBM always progresses due to its infiltrative nature and resistance to treatment.

### 4.2. Genomic Alterations in GBM

The genomic landscape of GBM is heterogeneous and involves many mutations, CNVs, and chromosomal rearrangements that drive tumorigenesis, progression, and invasion (Table 2). Mutations in TP53 are found in up to 60–70% of secondary GBMs and contribute to a loss of cell cycle control and apoptosis resistance [31]. Consequently, TP53 mutations result in an enhanced invasive ability due to the upregulation of EMT factors by the mutant p53 protein. Another common mutation is found within the EGFR, which is amplified and mutated in around 57% of primary GBM and 8% of secondary GBM cases, with the EGFRvIII variant being a common alteration that leads to constitutive receptor activation [32]. The difference between EGFR and EGFRvIII is that the latter is a constitutively active mutant variant causing uncontrolled growth behavior in the tumor. EGFR amplification enhances invasiveness by activating downstream pathways that promote proliferation, survival, and most importantly, invasion. The enhanced invasive nature of GBM is a result of upregulated matrix metalloproteinases that degrade the ECM, allowing tumor cells to spread to nearby brain tissue. Mutations in IDH1 (~80% of grade II-III gliomas and secondary GBMs and ~5% of primary GBMs) and less frequently IDH2 are characteristic of secondary GBM and are associated with a better prognosis [28,33]. Normally, IDH enzymes convert isocitrate to alpha-ketoglutarate as part of cellular metabolism. However, mutations in IDH1/2 disrupt the natural process, leading to the buildup of a cytotoxic metabolite that interferes with normal cellular functioning and causes genomic instability [33]. Oddly enough, wild-type IDH in GBM increases invasiveness by promoting angiogenesis and interacting with other tumorigenic pathways that facilitate tumor invasion.

CNVs and chromosomal rearrangements play a pivotal role in the genetic landscape of GBM, contributing to the complexity of this malignant brain tumor. Deletions of CDKN2A/B are prevalent and result in a loss of tumor suppressor functions, facilitating uncontrolled cell division. CDKN2A/B are tumor suppressor genes that produce proteins that inhibit cyclin-dependent kinases (CDKs) and are crucial for cell cycle regulation. The loss of these inhibitors in GBM can enhance tumor cell migration, contributing to the aggressive nature of this tumor. Gains of chromosome 7 (involving EGFR) and losses of chromosome 10 (involving PTEN) are hallmarks of GBM, leading to enhanced tumor cell proliferation, survival, and invasion, ultimately contributing to the rapid growth and poor prognosis of GBM. Among these, mutations and amplifications in EGFR are particularly prevalent, occurring in approximately 50% of total GBM cases [32]. EGFR alterations drive aberrant cell proliferation and survival by activating downstream pathways such as RAS/RAF/MEK/ERK and PI3K/AKT, which confer resistance to therapies and promote tumor growth. In contrast, PTEN, mutated in about 30–40% of cases, normally functions as a tumor suppressor by inhibiting the PI3K/AKT pathway [32]. A loss of PTEN function leads to constitutive pathway activation, enhancing cell survival and growth while impairing responses to apoptosis-inducing treatments. Less frequently seen are TERT promoter mutations, which lead to increased telomerase activity, enabling cells to maintain telomere length and proliferate indefinitely [34]. These genetic alterations collectively emphasize the complex molecular landscape of GBM and highlight potential targets for more effective therapeutic interventions aimed at improving patient outcomes.

### 4.3. Invasion in GBM

Invasion in GBM is a defining feature of this highly aggressive brain tumor that significantly complicates treatment and contributes to its poor prognosis. GBM cells possess the ability to infiltrate surrounding brain tissue, often migrating far from the primary tumor site into critical areas of the brain, which makes complete surgical resection impossible. This invasive behavior is driven by multiple molecular mechanisms, including the overexpression of MMPs and other enzymes that degrade the ECM, facilitating movement through the brain tissue. This degradation facilitates tumor cell movement throughout the tissue architecture of the brain. Signaling pathways are also frequently activated in GBM, encouraging cell survival, motility, and invasion. Additionally, complex genomic changes involving genetic and epigenetic alterations disrupt normal cellular functions and enhance the tumor cells’ invasive capabilities. These epigenetic modifications influence the expression of genes associated with invasion and resistance to treatment.

One protein of particular interest in the context of early tumorigenesis of GBM, AJAP1 (Adherens Junctions Associated Protein 1), is essential for maintaining cell adhesion and controlling motility, both of which are critical in the invasive nature of GBM cells [35]. In GBM, AJAP1 levels are frequently reduced or lost, disrupting normal cell adhesion and enhancing tumor cells’ mobility and invasiveness. There is a negative correlation between AJAP1 and EGFR expression: as AJAP1 decreases, EGFR activity increases, promoting aggressive tumor behavior. When there is an activation of the EGFR pathway, AJAP1 expression is suppressed via a PI3K/AKT-mediated mechanism, resulting in actin cytoskeleton remodeling and enhanced invasion [36]. Inhibition of the AKT pathway has shown potential in reversing the downregulation of AJAP1 in EGFRvIII-positive GBMs, thereby reducing invasiveness and potentially improving patient outcomes. However, this interplay between AJAP1 and EGFR complicates treatment and worsens the prognosis of GBM, highlighting the significant challenges in developing effective therapeutic interventions.

Another significant pair of proteolytic enzymes that play a crucial role in the invasion and migration of GBM are Urokinase Plasminogen Activator (uPA) and ADAMs (A Disintegrin and Metalloproteinases). By converting plasminogen to plasmin, uPA breaks down the ECM and promotes tumor cell migration and angiogenesis [37]. In individuals with GBM, elevated levels of uPA and its receptor are associated with increased invasiveness and poor prognosis. Similarly, ADAMs, particularly ADAM17, cleave ECM components and cell surface proteins, enhancing cell migration and invasion [37]. When overexpressed, ADAMs are linked to a higher degree of tumor aggressiveness. Together, these genomic factors create a tumor microenvironment conducive to invasion, complicating treatment and contributing to the poor prognosis associated with gliomas.

### 4.4. Tumor Microenvironment in GBM

The TME in GBM is a non-static, dynamic entity that evolves in response to therapeutic interventions and hypoxic conditions to facilitate invasion, with interactions with surrounding stromal cells, ECM components, and soluble factors such as cytokines promoting further invasion and aiding in the colonization of new sites [22,38,39]. The drastic differences in the microenvironment of GBM compared to other tumors contribute to tissue-specific outcomes and impact responses to therapy (Figure 4). For instance, while the TME in ovarian cancer may predominantly involve immune cell interactions and ascitic fluid dynamics, the GBM microenvironment is highly specialized, with its blood–brain barrier (BBB) posing an additional challenge for drug delivery [22]. This feature makes it difficult for many conventional therapies to penetrate the tumor and be effective, highlighting how the unique features of the TME impact treatment outcomes.

The genomic landscape of GBM is characterized by a range of alterations in key oncogenes and tumor suppressor genes, contributing to its aggressive nature and poor prognosis. It comprises diverse cellular components, including cancer cells, glioma stem cells, immune cells, astrocytes, and endothelial cells [38]. These cells interact within an acellular matrix rich in extracellular proteins, cytokines, growth factors, and metabolic factors, creating an environment that supports tumor proliferation, invasion, and immune evasion. Key features of the TME include abnormal vasculature leading to hypoxia, which further promotes tumor aggression by inducing hypoxia-inducible factors (HIFs) that upregulate genes associated with invasion and angiogenesis [13,38]. The immunosuppressive nature and metabolic adaptations of the TME of the brain contribute to therapeutic resistance, making it a critical target for developing more effective GBM treatments.

These differences in the microenvironment, compared to other cancers like ovarian cancer, result in unique challenges when it comes to treatment, highlighting how tissue-specific factors drive GBM’s aggressive behavior and influence therapeutic outcomes. For example, while VEGF inhibitors may be effective in inhibiting angiogenesis in other cancers, they have limited effectiveness in GBM due to the complex nature of its blood–brain barrier and abnormal vasculature [22].

Recent developments in genomic technologies have elucidated the complex relationship between genetic alterations and the TME in GBM. Single-cell sequencing has revealed discrete cellular subpopulations with different genetic and phenotypic features within GBM [40]. The behavior of these subpopulations can tremendously differ in terms of invasive ability, growth rates, and therapeutic response. Treatments may be less effective against different subpopulations because they may contain unique genetic mutations and alterations in signaling pathways. Furthermore, glioma stem cells, which reside in a specific niche within the TME, are notoriously resistant to conventional therapies and may be a significant source of recurrence.

As a result of the tumor’s heterogeneity, while some treatments may initially slow progression in GBM, resistant subpopulations will proliferate, leading to tumor recurrence and treatment failure [22]. This feature highlights the need for personalized treatment approaches that accommodate individual tumors with diverse cellular and molecular changes within their heterogeneous TME.

## 5. Comparative Analysis

### Similarities in Genomic Changes Driving EMT in Ovarian Cancer and Invasion in GBM

Both ovarian cancer and GBM exhibit significant genomic alterations that drive crucial transitions, such as EMT in ovarian cancer and invasion in GBM (Table 3). These processes underlie tumor progression, metastasis, invasion, and the establishment of secondary tumors. Shared pathways between ovarian cancer and GBM highlight the standard oncogenic processes that drive the development and progression of these two distinct but similarly aggressive malignancies. Both cancers frequently show alterations in the PI3K/AKT pathway. In ovarian cancer, mutations in PIK3CA activate this pathway, promoting cell survival and proliferation. Similarly, in GBM, PTEN loss leads to PI3K/AKT pathway activation, enhancing tumor growth. Additionally, the activation of the RAS/MAPK pathway is common in both cancers, promoting cell proliferation and survival, thereby contributing to the aggressive nature of these tumors.

Common transcription factors and signaling molecules play significant roles in the progression of ovarian cancer and GBM, emphasizing their shared molecular mechanisms. Key transcription factors such as Snail, Slug, and Twist1 are implicated in EMT in ovarian cancer by repressing E-cadherin and promoting mesenchymal traits essential for metastasis. In GBM, similar mechanisms facilitate invasion through the downregulation of adhesion proteins like AJAP1 and the upregulation of proteolytic enzymes like MMPs, uPA, and ADAMs. Additionally, both cancers exhibit changes in signaling molecules like TGF-β, which is crucial for EMT induction in ovarian cancer and plays a significant role in GBM progression by promoting invasion. Understanding these shared transcription factors and signaling molecules highlights the commonalities in their oncogenic pathways and opens potential avenues for targeted therapies that could benefit patients suffering from these aggressive malignancies.

Differences in genomic alterations and their impacts on ovarian cancer and GBM reveal distinct molecular landscapes and disease behaviors. In ovarian cancer, BRCA1 and BRCA2 mutations are prevalent, leading to defective DNA repair and increased genomic instability. Additionally, TP53 mutations are ubiquitous in HGSC, resulting in the loss of cell cycle control. In contrast, GBM commonly exhibits EGFR amplification and mutations, particularly the EGFRvIII variant, which leads to continuous receptor activation and tumor growth [32]. Another characteristic of GBM is the presence of IDH1/2 mutations, typically found in secondary GBM, which are associated with a better prognosis and distinct metabolic changes [28].

Tissue-specific factors also play crucial roles in the progression of these cancers. In ovarian cancer, the peritoneal environment and the hormone-responsive nature of the disease significantly influence its progression and metastasis. The fallopian tube epithelium is often implicated as the origin of ovarian cancer [41]. Conversely, in GBM, the brain microenvironment, including interactions with neural and glial cells, shapes the tumor’s progression. The blood–brain barrier presents a unique challenge for treatment delivery, making GBM particularly difficult to treat [42]. Understanding these differences in tissue-specific factors is essential for developing targeted therapies and improving patient outcomes for both types of cancer.

Variations in the TME between ovarian cancer and GBM influence the behavior and progression of these malignancies. In ovarian cancer, the peritoneal fluid and omental fat provide a rich source of growth factors and cytokines, which are essential for cancer cell survival and dissemination [43]. This microenvironment supports the proliferation of tumor cells and their spread to other parts of the peritoneal cavity. Additionally, immune cells such as tumor-associated macrophages (TAMs) and CAFs play a crucial role in promoting EMT and metastasis [22]. These cells create a supportive niche that enhances the invasiveness and spread of ovarian cancer cells.

In contrast, the TME in GBM is shaped by the brain’s unique extracellular matrix and various stromal cells, including astrocytes and microglia. These cells create a supportive niche that allows GBM growth and progression. The immune response in GBM is notably characterized by an immunosuppressive milieu, which limits effective anti-tumor immunity [38]. This immunosuppressive environment is a significant barrier to the successful treatment of GBM, as it hampers the body’s natural ability to fight the tumor. Understanding the distinct TME variations in ovarian cancer and GBM is crucial for developing targeted therapies that can effectively disrupt these supportive niches and improve treatment outcomes.

Implications for treatment and prognosis in ovarian cancer and GBM highlight the importance of targeted therapies and potential biomarkers. In ovarian cancer, PARP inhibitors have shown effectiveness in BRCA-mutated cases by targeting the defective DNA repair pathway. Additionally, anti-angiogenic agents and PI3K/AKT inhibitors are under investigation, offering promising avenues for therapy [19]. For GBM, researchers are exploring EGFR inhibitors, IDH1/2 inhibitors, and therapies targeting the PI3K/AKT pathway. Immunotherapies, including checkpoint inhibitors, are also being studied; however, their efficacy has been limited by the immunosuppressive microenvironment of GBM [37,44,45]. Potential biomarkers for EMT and invasion further inform treatment strategies. In ovarian cancer, EMT biomarkers include E-cadherin downregulation, vimentin upregulation, and transcription factors like Snail and Twist1. Conversely, in GBM, invasion can be indicated by the overexpression of E-cadherin, downregulation of mesenchymal markers, and alterations in TGF-β signaling components. Identifying and targeting these biomarkers can aid in developing more effective treatments and improve prognosis for patients with these aggressive cancers.

**Figure 5:**
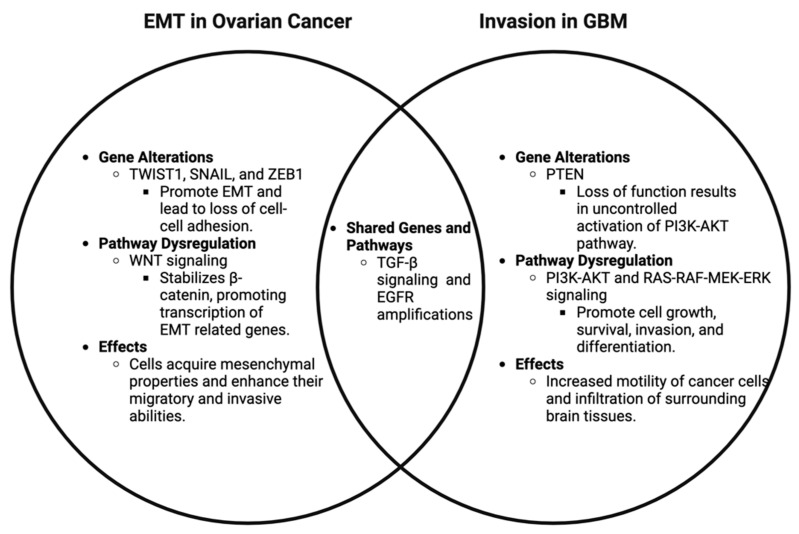
**Comparative analysis of invasion and EMT-related genomic alterations in ovarian cancer and GBM.** This Venn diagram presents a comparative analysis of genomic alterations related to invasion and EMT in ovarian cancer and GBM. The diagram shows unique genomic alterations associated with invasion and EMT specific to each GBM and ovarian cancer in the non-overlapping sections. The overlapping section illustrates shared genomic changes, highlighting common pathways and molecular mechanisms. This comparison underscores the similarities and differences in the genetic underpinnings of these aggressive behaviors in the two cancer types [46].

The comparative analysis of ovarian cancer and GBM highlights commonalities and differences in their genomic landscapes and the processes driving EMT and invasion, respectively (Figure 5). Understanding these similarities and differences is crucial for developing effective targeted therapies and improving prognosis. The TME and tissue-specific factors significantly shape these cancers’ progression and treatment response, emphasizing the need for tailored therapeutic approaches. Identifying reliable biomarkers for EMT and invasion can further enhance the precision of treatments and facilitate the early detection of metastatic potential.

## 6. Discussion

Comparative genomic studies reveal intriguing insights into the roles of EMT in ovarian cancer and invasion in GBM. These findings often highlight both commonalities and distinct differences in the genomic landscape of these cancers. For instance, while both cancers may exhibit alterations in EMT-related pathways, the specific genetic mutations or amplifications in EMT genes can vary significantly between ovarian cancer and GBM. Understanding these genomic profiles is crucial for pinpointing potential therapeutic targets and prognostic markers unique to each cancer.

Despite advances, several challenges hinder comprehensive understanding and effective targeting of EMT in ovarian cancer and invasion in GBM. Variability in EMT/invasion marker expression complicates clinical applicability, while the dynamic nature of these transitions during cancer progression necessitates longitudinal studies. Technical limitations in genomic profiling and single-cell analysis also impede the precise characterization of EMT/invasion states within heterogeneous tumor populations. Furthermore, deciphering context-specific roles of EMT/invasion in different microenvironments remains a critical challenge in translational research. For example, targeting TGF-β in ovarian cancer has shown promise in preclinical studies, where the inhibition of TGF-β signaling reduced EMT, inhibited tumor growth, and enhanced chemotherapy effectiveness [47,48]. TGF-β is particularly significant in ovarian cancer, as it contributes to the progression of both primary tumors and metastases, promoting immune evasion and resistance to therapy. TGF-β inhibitors have demonstrated the potential to reduce the spread of ovarian cancer cells, overcome chemotherapy resistance, and improve patient survival rates in preclinical models [48].

The early detection of genomic or molecular events associated with EMT can significantly improve cancer prognosis by enabling timely and targeted therapeutic interventions. Identifying these changes before the cancer has spread allows for more effective treatment strategies to prevent metastasis and/or invasion. This proactive approach improves treatment outcomes and enhances the overall survival rates for patients with cancer. Future research should elucidate context-specific regulators and signaling pathways driving EMT/invasion transitions in ovarian cancer and GBM. Integrative multi-omic approaches, including genomics, epigenomics, and proteomics, will be instrumental in uncovering novel biomarkers and therapeutic targets. Leveraging advanced imaging techniques and preclinical models that recapitulate the TME will provide deeper insights into spatial and temporal dynamics of EMT/invasion. Moreover, collaborative efforts to establish standardized EMT/invasion characterization protocols across clinical trials are essential for validating emerging biomarkers and therapeutic strategies.

Emerging therapeutic approaches targeting EMT and invasion hold promise for ovarian cancer and GBM treatment. Strategies include small-molecule inhibitors of EMT-inducing transcription factors or signaling pathways, such as TGF-β or Wnt/β-catenin, to prevent EMT initiation or maintenance [49]. Combination therapies targeting EMT/invasion in ovarian cancer and GBM, alongside conventional treatments such as chemotherapy, radiation, or immunotherapy, have shown the potential to improve patient outcomes. For example, TGF-β inhibitors in ovarian cancer have enhanced chemotherapy response and reduced metastasis [47,48]. Personalized medicine approaches based on individual EMT/invasion profiles may optimize therapeutic efficacy and minimize treatment resistance in ovarian cancer and GBM. While challenges persist, ongoing research into EMT in ovarian cancer and invasion in GBM holds considerable promise for advancing our understanding of cancer biology and improving clinical outcomes through targeted therapeutic interventions.

## 7. Conclusions

In conclusion, the comparative genomic analysis of EMT in ovarian cancer and invasion in GBM offers valuable insights into the underlying molecular mechanisms driving these aggressive malignancies. Through this analysis, we have gleaned nuanced differences and commonalities in the genomic alterations associated with EMT/invasion across ovarian cancer and GBM. While both cancer types exhibit dynamic transitions between epithelial and mesenchymal states, the specific genetic landscapes governing these transitions vary significantly. This understanding underscores the importance of tailored therapeutic strategies that account for the unique molecular profiles of each cancer.

The significance of EMT/invasion in ovarian cancer and GBM cannot be overstated. EMT enables cancer cells to acquire invasive and metastatic properties, contributing to disease progression and therapeutic resistance. By targeting these transition states, clinicians and researchers can potentially disrupt key drivers of metastasis and improve patient outcomes.

Integrating genomic, transcriptomic, and proteomic data will be crucial for unraveling the development of EMT/invasion in cancer progression. Advanced technologies and computational tools will aid in identifying novel biomarkers and therapeutic targets, paving the way for personalized treatment approaches tailored to individual patient profiles. Moreover, collaborative efforts across disciplines and institutions will be essential to validate findings and translate discoveries into clinical practice. In conclusion, the comparative genomic analysis of EMT/invasion in ovarian cancer and GBM enhances our understanding of cancer biology and holds profound implications for cancer treatment and research. By elucidating the molecular mechanisms driving EMT/invasion transitions, we can forge new paths toward more effective therapies and ultimately improve outcomes for patients battling these challenging diseases.

## Figures and Tables

**Table 1:** Summary of genomic alterations in ovarian cancer.

Genomic Alteration	Pathway Affected	Frequency	Function
TP53 Mutations	DNA Repair, Cell Cycle	~96% of HGSC	Disrupts tumor suppression, causes genomic instability, and fuels cancer progression
BRCA1/BRCA2 Mutations	DNA Repair	5–15% of all subtypes of ovarian cancer	Messes up DNA repair, makes tumors more sensitive to platinum-based treatments and PARP inhibitors
PIK3CA Mutations	PI3K/AKT Pathway	Common in endometroid and clear cell types	Causes unchecked cell growth and survival and makes it harder for cells to die off
PTEN Deletions	PI3K/AKT Pathway	12–20% of all subtypes of ovarian cancer	This deletion leads to uncontrolled cell growth and survival and makes it challenging for treatments to work
KRAS Mutations	RAS/MAPK Pathway	10–15% of all subtypes of ovarian cancer	Keeps the growth and survival pathways constantly on, promoting tumor growth
CCNE1 Amplifications	DNA Repair, Cell Cycle	Commonly seen in cases	Causes replication stress and instability, linked to poor prognosis
CDKN2A/B Deletions	Cell Cycle	Commonly seen in cases	This deletion leads to loss of cell cycle control, causing uncontrolled cell growth

**Table 2:** Summary of genomic alterations in GBM.

Genomic Alteration	Frequency	GBM Type	Impact
TP53 Mutation	60–70%	Secondary GBM	This mutation leads to cell cycle regulation disruption and cell death resistance, resulting in increased invasiveness by promoting EMT factors by mutant p53.
EGFR Mutation	57% in primary GBM, 8% in secondary GBM	Both	Causes continuous activation of the receptor, fostering tumor growth, cell survival, and movement. The EGFRvIII variant contributes to the tumor’s aggressive nature, enhances matrix metalloproteinase activity, and triggers EMT.
IDH1/IDH2 Mutation	5–10%	Secondary GBM	Correlated with a more favorable prognosis. In contrast, wild-type IDH promotes new blood vessel formation and supports tumor invasion.
CDKN2A/B Deletion	Prevalent	Both	Results in the loss of tumor-suppressing capabilities, leading to unchecked cell division and increased tumor cell movement, contributing to the tumor’s aggressive characteristics.
EGFR Amplification	~50%	Both	It drives abnormal cell growth and survival, activating pathways like RAS/RAF/MEK/ERK and PI3K/AKT, leading to therapy resistance.
PTEN Mutation	30–40%	Both	Loss of function as a tumor suppressor, leading to constant activation of the PI3K/AKT pathway, promoting cell survival and growth while reducing response to treatments that induce cell death.
TERT Promotor Mutation	Less frequent	Both	Increases telomerase activity, allowing cells to maintain telomere length and continue dividing indefinitely.

**Table 3:** Key transcription factors involved in EMT in ovarian cancer and invasion in GBM.

Transcription Factor	Role in EMT/Invasion	Associated Genomic Changes	Cancer Type
Snail (SNAI1)	Induces EMT by repressing E-cadherin	Upregulation leads to loss of E-cadherin expression	Ovarian, GBM
Slug (SNAI2)	Promotes EMT by downregulating epithelial markers	Upregulation and promoter hypermethylation	Ovarian, GBM
Twist1	Initiates EMT, promotes cell invasion	Gene amplification, upregulation	Ovarian, GBM
Zeb1	Represses epithelial markers, induces mesenchymal markers	Gene amplification, increased expression	Ovarian, GBM
Zeb2	Like Zeb1, promotes EMT	Increased expression	Ovarian, GBM
E-cadherin (CDH1)	Maintains epithelial phenotype, inhibits EMT	Downregulation or loss of function mutations	Ovarian, GBM
FOXC2	Promotes EMT, involved in metastasis	Upregulation	Ovarian
Sox4	Induces EMT, promotes cell migration	Increased expression	GBM
YB-1	Enhances EMT by regulating Snail and Twist	Upregulation	GBM

## Data Availability

Not applicable.
